# Expanding HIV Prevention: Exploring Community Pharmacists’ Willingness to Provide PrEP in Nigeria

**DOI:** 10.1007/s10461-025-04923-4

**Published:** 2025-10-18

**Authors:** Theodora C. Omenoba, Ugochi Eyong, Valentine Okelu, Tyler Nauta, Amarachi Nwafor, Ambrose Eze, Chimezie Anyakora, Obinna I. Ekwunife

**Affiliations:** 1 Department of Clinical Pharmacy and Pharmacy Management, Veritas University, Abuja, Nigeria; 2 Bloom Public Health, Abuja, Nigeria; 3 Department of Clinical Pharmacy and Pharmacy Management, Nnamdi Azikiwe University, Agulu, Nigeria; 4 Department of Medicine, University at Buffalo, New York, USA; 5 Lone Star Community College, Houston, TX USA; 6 Association of Community Pharmacists of Nigeria, Lagos, Nigeria; 7 School of Science and Technology, Pan-Atlantic University, Lagos, Nigeria; 8 Division of Population Health, Department of Medicine, University at Buffalo, New York, USA

**Keywords:** Community pharmacists, Pre-exposure prophylaxis (PrEP), Willingness to provide PrEP, Key populations, Nigeria

## Abstract

HIV remains a significant public health challenge in Nigeria. Pre-exposure prophylaxis (PrEP) effectively prevents HIV, but uptake is hindered by stigma, limited clinic access, and provider shortages. Community pharmacies could expand access, but their role in PrEP delivery remains underexplored. We assessed Nigerian community pharmacists’ willingness and support needs for delivering pharmacy-delivered oral PrEP, examined association between sociodemographic factors and willingness, and compared pharmacy geolocations with sex worker hotspots and hospitals providing PrEP. A descriptive cross-sectional survey was conducted among licensed community pharmacists in Abuja and Lagos. Data collection was conducted using a hybrid approach (on-line and on-site) over two months from January to March 2025. Associations between sociodemographic factors and willingness to provide PrEP were examined using chi-square and Fisher’s exact tests. ArcGIS distance analysis compared pharmacy geolocations with sex worker hotspots and hospitals offering PrEP. Of 267 pharmacists, 99.6% expressed willingness to deliver PrEP. Key support needs included PrEP guidelines training (92.1%), access to HIV rapid tests (81.3%), counseling skills (70.8%), and logistical support (70.8%). Structural readiness was high, with significant gender differences in willingness to serve gay, bisexual, and men who have sex with men (male: 85.6% vs. female: 67.3%; χ^2^ (1) = 0.22, *p* = 0.001). In Abuja and Lagos, 82.5% and 93.5% of key population hotspots, respectively, were closer to pharmacies than hospitals (*p* < 0.0001). Nigerian pharmacists are highly willing to provide PrEP. Training, particularly in gender-sensitive stigma reduction, and logistical support are essential for successful implementation.

## Introduction

HIV remains a significant public health challenge in Nigeria, with an estimated 2 million people living with HIV [1]. The adult HIV prevalence rate is 1.3%, with 47 new infections per 100,000 people yearly, and the highest concentrations occuring in the South-South (3.1%), North-Central (2.1%) and South-East regions (1.9%) [2], underscoring the urgent need for innovative prevention and treatment strategies [1]. When taken consistently, daily oral pre-exposure prophylaxis (PrEP) with emtricitabine/tenofovir disoproxil fumarate (FTC/TDF) is approximately 99% effective in preventing HIV acquisition [3–6]. Although this study focuses on daily oral PrEP, long-acting options are also becoming available. For instance, long-acting injectable cabotegravir (CAB-LA), approved by the US Food and Drug Administration (FDA) offers protection for up to two months with a single dose [7]. In addition, intravenous, subcutaneous, implants, and transdermal delivery systems, as well as extended duration oral drugs are promising formulations being developed to enhance adherence and expand prevention options [8, 9]. These long-acting options offer greater flexibility and may improve adherence among individuals facing challenges with daily oral medication [10, 11], although specialized training for administration could limit feasibility in pharmacies [12]. While oral PrEP is effective, its uptake remains suboptimal due to several barriers, including stigma, limited access to healthcare facilities, restrictive clinic hours, and inadequate awareness among potential users [3, 13, 14].

Community pharmacies are well-positioned to expand access to PrEP due to their accessibility, convenience, trustworthiness, and role as first-line healthcare providers in both rural and urban areas [15–17]. Community pharmacies are retail outlets managed by licensed pharmacists that provide medications, health counseling, and other pharmaceutical services. Pharmacy-based PrEP delivery models in the United States and Kenya have demonstrated success in improving access and uptake of HIV prevention services while addressing stigma and increasing convenience [17–19]. In Nigeria, PrEP is predominantly offered in public hospitals, specialized HIV clinics, and selected non-governmental organizations facilities [20]. Private community pharmacies are licensed to sell pre-packaged, over-the-counter and prescription medicines and have historically delivered sexual and reproductive health services to people [21, 22]. This suggests that integrating PrEP into community pharmacies could significantly enhance access to PrEP, particularly for key populations and young people who may face discrimination in traditional healthcare settings.

Additionally, retail pharmacies are widespread in Nigeria and often located in areas where sex work is prevalent making them convenient access points for PrEP [23]. This co-location is important given the disproportionately high HIV prevalence among key populations, including 15.5% among female sex workers, 25.0% among men who have sex with men, and 28.8% among transgender people, compared with 1.3% in the general adult population [24]. While pharmacy-based PrEP delivery may present challenges, formative studies have offered recommendations to enhance its implementation, including pharmacist education and training, eligibility screening, logistics monitoring, confidentiality safeguards, reimbursement mechanisms, increased awareness, and referral linkages [17, 25, 26]. However, successful implementation ultimately depends on pharmacists’ willingness and active participation [27]. For example, a study in Nebraska and Iowa found that although pharmacists had limited familiarity with PrEP and CDC guidelines, they expressed high interest in providing PrEP services [27]. A similar finding was reported by Yoong et al. [28]. In Nigeria, a study exploring the potential expansion of community pharmacists’ roles in PrEP delivery revealed knowledge gaps in treatment guidelines, medication management, and counseling skills [29]. Moreover, little evidence exists on pharmacists’ support needs to take on this expanded role in Nigeria.

Therefore, this study assessed the willingness and support needs of community pharmacists in Abuja and Lagos State, Nigeria, to provide PrEP services. It also mapped the spatial distribution of pharmacies expressing interest in PrEP delivery and compared their proximity to sex work hotspots with that of existing hospitals offering PrEP services. These findings aim to inform strategies for implementing a pharmacy-based PrEP delivery model to strengthen HIV prevention efforts in Nigeria.

## Methods

### Study Design and Setting

This descriptive, cross-sectional study was conducted among licensed community pharmacists practicing in two major cities in Nigeria: Lagos State and the Federal Capital Territory (FCT), Abuja. These cities were purposively selected due to their high HIV burden and concentration of community pharmacies [30, 31]. Data were collected as part of an initiative to explore the willingness and support needs of community pharmacists to deliver pre-exposure prophylaxis (PrEP) services. According to Pharmacy Council of Nigeria records, as of December 2024, there were 1741 licensed community pharmacies in Lagos State and 937 in FCT, Abuja [32].

### Study Population and Sampling

The study population comprised of licensed community pharmacists. Participants were selected using a purposive sampling method during professional events, conferences, and on-site visits to community pharmacies. Additionally, online recruitment was conducted via Association of Community Pharmacists of Nigeria (ACPN)-affiliated WhatsApp platforms. While this sampling approach allowed us to reach a substantial number of the ACPN members in FCT Abuja and Lagos, it may limit generalizability to those who are less engaged in its activities. Eligibility criteria included active licensure, current practice in a community pharmacy setting, and willingness to provide informed consent.

### Data Collection Tool and Procedure

Data were collected using a structured, pre-tested questionnaire. The questionnaire items were generated based on an adapted Theory of Planned Behavior (TPB) [33], under the premise that a more favorable attitude toward PrEP delivery and a higher perceived ability to provide the service would be associated with greater willingness among community pharmacists to offer PrEP. While the TPB served as a conceptual framework for item generation, this study did not conduct formal hypothesis testing or statistical modeling to evaluate the theory. Item development was also informed by evidence from prior implementation studies on pharmacy-based PrEP delivery, particularly from Kenya and the United States [17, 34]. To enhance contextual validity, draft items were reviewed and refined through consultations with public health stakeholders and practicing pharmacists in Nigeria [26]. The final questionnaire captured constructs derived from TPB, including attitudes, perceived control, and intention, alongside contextual factors relevant to the Nigerian pharmacy setting. The specific items generated from these theoretical constructs are summarized in Table 1. Additionally, the questionnaire included items on socio-demographic characteristics (e.g., gender, years of practice, professional affiliations), as well as awareness and sources of information about PrEP. Geolocation data of respondents’ pharmacies were collected to enable spatial mapping of pharmacies interested in provision of PrEP service. Most questions used yes/no/not sure response options, with “not sure” responses treated as “no” during analysis. Data collection was conducted over two months from January to March 2025. Questionnaires were administered in person during professional gatherings and routine visits to community pharmacies. Additionally, electronic surveys were distributed through pharmacists’ WhatsApp platforms via a secure link generated using KoboToolbox. Follow-up reminders were sent through the same platform to improve response rates. Informed consent was obtained from all participants prior to survey completion.

**Table 1:** Items generated based on theory of planned behavior

Applicable constructs theory of planned behavior	Definition of construct	Items generated
Attitude	Personal evaluation of offering PrEP service	Do you think delivering PrEP through pharmacies is an effective strategy for reducing HIV transmission?
Perceived Control	Ability to offer PrEP service/perceived facilitators and barriers	Do you have a private consulting room in your pharmacy? What additional support or training would you need to offer PrEP services? Will you like the assistance of a nurse or a HIV certified tester to support the service? Would you be willing to work with healthcare facilities for referrals of HIV-positive clients or consult a HIV clinician in case you need clarification while offering service?
Intention	Willingness to offer the service	Would you be willing to offer PrEP services to sex workers? Would you be willing to offer PrEP services to Men who have sex with Men? Would you be willing to offer PrEP services to adolescents? Would you be willing to stock PrEP medication in your Pharmacy?

A map was created for Lagos and Abuja using ArcGIS Pro 3.4, indicating the locations of hospitals delivering PrEP services [35, 36], pharmacies interests in providing PrEP services, and areas identified as sex work hotspots. While some public health mapping reports provided general categories of sex work hotspots that exist such as hotels, brothels, street and nightclubs [30, 37], we additionally identified hotspot locations through supplementary sources including verified blog posts [38–40]. A 2.5 km buffer was applied around each point to represent a walkable distance (approximately 30 min one-way or a 1-hour round-trip). This spatial overlay was used to visualize geographic clusters of need relative to available resources.

### Study Outcomes

The primary outcomes were pharmacists’ willingness to deliver PrEP services (measured using multiple willingness items) and their perceived support needs for PrEP delivery. Secondary outcomes included awareness of PrEP, availability of private consultation rooms, and the relative proximity of pharmacies to sex work hotspots compared with hospitals offering PrEP. Spatial accessibility was assessed by measuring the distance from each identified hotspot to the nearest pharmacy or hospital.

### Data Analysis

Data were analyzed using SPSS version 22.0. Descriptive statistics (frequencies and percentages) were used to summarize participant characteristics and responses. Chi-square tests (or Fisher’s exact test, where appropriate) were used to assess associations between respondent characteristics (e.g., gender and years of experience) and willingness to deliver PrEP services. Statistical significance was set at *p* < 0.05.

For the spatial analysis, GPS coordinates of hospitals, pharmacies, and sex work hotspots in Lagos and Abuja were analyzed using ArcGIS Pro 3.4. The nearest facility function was used to calculate the distance from each hotspot to the closest hospital or pharmacy. Accessibility was assessed by comparing mean distances (with standard deviations) to each facility type and by determining the proportion of hotspots located closer to a pharmacy versus a hospital. Differences in mean distance were quantified using standardized mean difference (SMD) and tested for statistical significance with an independent two-tailed t-test in Microsoft Excel (version 2502). This analysis provided insights into the accessibility of HIV PrEP services for high-need populations and identified areas where pharmacy-based PrEP delivery could have the greatest impact.

## Results

### Sociodemographic Characteristics of Participants

A total of 267 community pharmacists participated in the study, with majority being male (58.5%, *n* = 155) (Table 2). Most participants had over 10 years of experience practicing as pharmacists (62.9%, *n* = 168), followed by those with 5–10 years of experience (26.2%, *n* = 70), and those with less than 5 years of experience (10.9%, *n* = 29). A large proportion of participants (85.4%, *n* = 228) reported being active members of the ACPN, with current membership and/or active involvement in ACPN activities. Regarding awareness of PrEP, most participants (94.4%, *n* = 252) were aware of HIV pre-exposure prophylaxis (PrEP) as a method of HIV prevention. Among those who were aware of PrEP, the most common source of awareness was professional training (80.9%, *n* = 216), followed by conferences (44.9%, *n* = 120), media (32.2%, *n* = 86), and other sources (7.1%, *n* = 19).

**Table 2:** Sociodemographic characteristics of study participants (*N* = 267)

Variable	Frequency (%)^a^
Sex	
Male	155 (58.5%)
Female	110 (41.5%)
Year of practice as a pharmacist	
< 5 years	29 (10.9%)
5–10 years	70 (26.2%)
> 10 years	168 (62.9%)
An active member of ACPN	
No	39 (14.6%)
Yes	228 (85.4%)
Awareness of HIV PrEP as an HIV prevention method	
No	15 (5.6%)
Yes	252 (94.4%)
How respondents became aware of HIV PrEP	
Professional training	216 (80.9%)
Conferences	120 (44.9%)
Media	86 (32.2%)
Other	19 (7.1%)

*PrEP* Pre-exposure prophylaxis

^a^Frequency may not sum up to 267 because of missing values

### Willingness to Offer PrEP Services Within the Pharmacy Setting

All respondents (100%) perceived pharmacy-based PrEP delivery as an effective HIV prevention strategy (Table 3). Nearly all (99.6%) expressed willingness to provide PrEP services at their pharmacy. The most reported support needs included training in PrEP guidelines (92.1%), provision of free HIV rapid diagnostic test (RDT) kits (81.3%), logistical support (70.8%), and counseling skills (70.8%). Only 3 pharmacies (1.1%) lacked a private consulting room, indicating that the majority (98.1%) had the necessary infrastructure for confidential client engagement. Additionally, 83.9% of respondents expressed interest in support from a nurse or HIV-certified tester/counselor to aid in PrEP service delivery.

**Table 3:** Willingness to deliver pharmacy-based PrEP and perceived support needs (*N* = 267)

Variable	Frequency (%)^a^
Perception of PrEP delivery through pharmacies as an effective HIV prevention strategy	
Effective	267 (100.0%)
Not effective	0 (0.0%)
Willingness to provide PrEP services at own pharmacy	
Willing	266 (99.6%)
Not willing	1 (0.4%)
Support or training needed to deliver PrEP	
Training in PrEP guidelines	246 (92.1%)
Provision of free HIV RDT kits	217 (81.3%)
Logistic support	189 (70.8%)
Counselling skills	189 (70.8%)
Others	8 (3.0%)
Availability of private consulting room in the pharmacy	
No	3 (1.1%)
Yes	262 (98.9%)
Willingness to receive support from a nurse or HIV-certified tester/counselor for PrEP service delivery	
Willing	224 (84.8%)
Not willing	40 (15.2%)

*PrEP* pre-exposure prophylaxis, *RDT* rapid diagnostic test

^a^Frequency may not sum up to 267 because of missing values. Percentages are based on the number of responses per item. Two responses were missing for the consultation room item; three were missing for interest in nurse/counselor support

### Willingness to Offer PrEP Services to Specific Groups by Respondent Demographics

The association between respondents’ characteristics and willingness to deliver pharmacy-based PrEP services is shown in Table 4. Willingness to collaborate with a health facility for PrEP delivery was high across both genders (96.0% in males vs. 93.5% in females; χ^2^(df) = 0.83 (1), *p* = 0.26) and did not significantly vary by years of experience (χ^2^(df) = 1.00 (2), *p* = 0.60). Willingness to offer PrEP to sex workers was similarly high across all groups (95.5% in males and 94.5% in females), with no significant association by gender (χ^2^(df) = 0.13 (1), *p* = 0.47) or years of experience (χ^2^(df) = 2.48 (2), *p* = 0.29). However, a statistically significant association was observed between gender and willingness to offer PrEP to gay, bisexual, and other men who have sex with men (GBMSM). Male respondents (85.6%) were more likely to express willingness to offer PrEP to GBMSM compared to female respondents (67.3%) (χ^2^(df) = 0.22 (1), *p* = 0.001). No significant association was found between willingness to offer PrEP to GBMSM and years of experience (χ^2^(df) = 0.97 (2), *p* = 0.62). Willingness to be assisted by a nurse or HIV-certified tester/counselor was generally high and did not differ significantly by gender (χ^2^(df) = 9.82 (1), *p* = 0.78) or years of experience (χ^2^(df) = 1.94 (2), *p* = 0.38).

**Table 4:** Association between respondent characteristics and willingness to deliver pharmacy-based PrEP (*N* = 267)

Characteristic	Category	Willing, *n* (%)	Not willing, *n* (%)	Total^a^ (*n*)	χ^2^ (df)	*p* value
Willingness to collaborate with a health facility for PrEP delivery						
Gender	Male	145 (96.0%)	6 (4.0%)	151	0.83 (1)	0.26
	Female	101 (93.5%)	7(6.5%)	108		
Years of experience	< 5 years	25 (92.6%)	2 (7.4%)	27	1.00 (2)	0.60
	5–10 years	66 (97.1%)	2 (2.9%)	68		
	> 10 years	157 (94.6%)	9 (5.4%)	166		
Willingness to offer PrEP to sex workers						
Gender	Male	147 (95.5%)	7 (4.5%)	154	0.13 (1)	0.47
	Female	103 (94.5%)	6 (5.5%)	109		
Years of experience	< 5 years	25 (89.3%)	3 (10.7%)	28	2.48 (2)	0.29
	5–10 years	68 (97.1%)	2 (2.9%)	70		
	> 10 years	158 (94.6%)	9 (5.4%)	167		
Willingness to offer PrEP to GBMSM						
Gender	Male	131 (85.6%)	22 (14.4%)	153	0.22 (1)	0.001*
	Female	74 (67.3%)	36 (32.7%)	110		
Years of experience	< 5 years	20 (69.0%)	9 (31.0%)	29	0.97 (2)	0.62
	5–10 years	54 (77.1%)	16 (22.9%)	70		
	> 10 years	129 (77.2%)	38 (22.8%)	167		
Willing to be assisted by a nurse or HIV-certified tester/counselor to support PrEP delivery						
Gender	Male	129 (83.8%)	25 (16.2%)	154	9.82 (1)	0.78
	Female	91 (83.5%)	18 (16.5%)	109		
Years of experience	< 5 years	24 (85.7%)	4 (14.3%)	28	1.94 (2)	0.38
	5–10 years	55 (79.7%)	14 (20.3%)	69		
	> 10 years	145 (86.8%)	22 (13.2%)	167		

*PrEP* pre-exposure prophylaxis, *RDT* rapid diagnostic test, *GBMSM* gay, bi-sexual and Men who have sex with men

^a^Total may not sum up to 267 because of missing values. Percentages are based on the number of responses per item

*Statistically significant

### Spatial Distribution of Pharmacies Interested in Offering PrEP Services

**Fig. 1:**
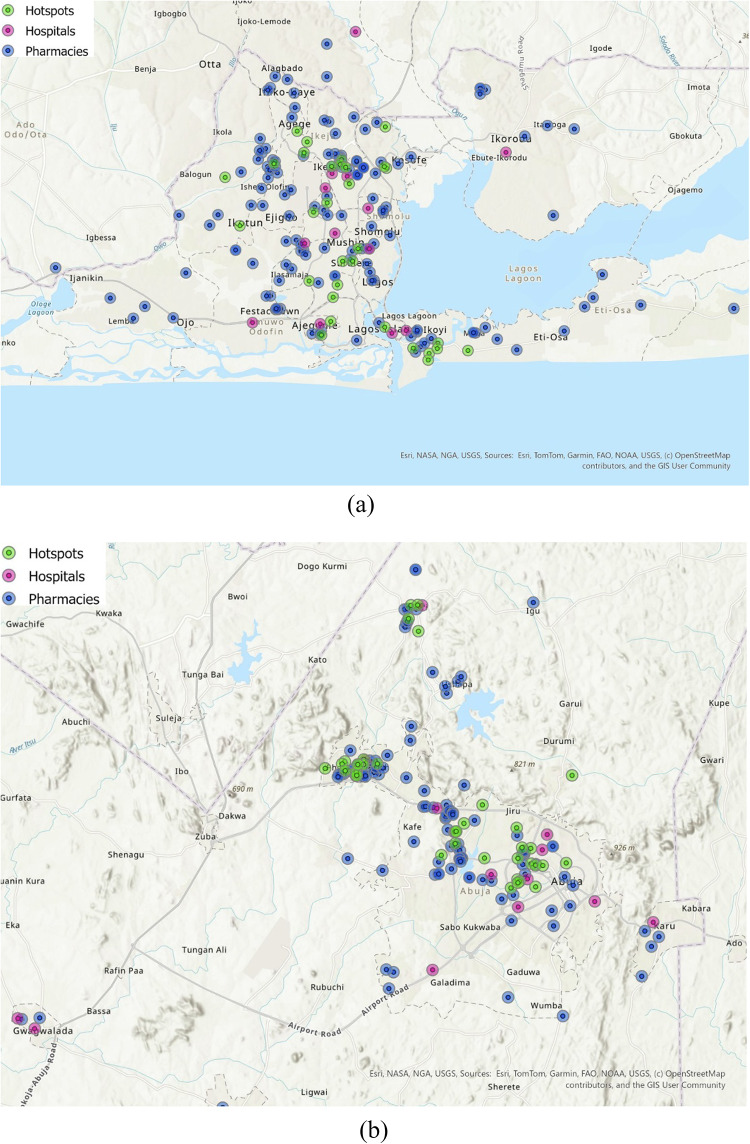
Map of **a** Abuja and **b** Lagos showing the proximity of pharmacies interested in PrEP delivery and hospitals providing PrEP services to identified sex work hotspots

The spatial distribution of pharmacies interested in offering PrEP services and hospitals in Abuja and Lagos is shown in Fig. 1. The majority of sex work hotspots were located closer to a pharmacy than to a hospital—33 hotspots in Abuja (82.5%) and 29 hotspots in Lagos (93.5%) were nearer to a pharmacy. In Abuja, the mean distance from hotspots to hospitals was 1836 m (SD = 154 m), compared with 770 m (SD = 1027 m) for pharmacies, yielding a standardized mean difference (SMD) of 1092 m. In Lagos, the mean distance to hospitals was 3241 m (SD = 2156 m) compared to 875 m (SD = 597 m) for pharmacies, with an SMD of 1582 m (Table 5).

**Table 5:** Accessibility of key population hotspots to hospitals and pharmacies

Accessibility to facility	Distance in meters (standard deviation) or frequency (%)	Standardized mean difference	*p* value
Abuja			
Key population hotspots mean distance from facility			
Hospital	1836.64 m (1154.45 m)	1092.64	< 0.0001
Pharmacy	770.62 m (1027.12 m)		
Key population hotspots closest to facility			
Hospital	7 (17.5%)		
Pharmacy	33 (82.5%)		
Lagos			
Key population hotspots mean distance from facility			
Hospital	3241.56 m (2156.52 m)	1582.26	< 0.00001
Pharmacy	875.38 m (597.09 m)		
Number of key population hotspots closest to facility			
Hospital	2 (6.45%)		
Pharmacy	29 (93.5%)		

## Discussion

This study highlights several important findings. Notably, some Nigerian pharmacists (i.e., those that accepted to participate in the study) are interested in offering PrEP services. These pharmacists acknowledged the need for training on PrEP guidelines, provision of free HIV rapid diagnostic test (RDT) kits, logistical support to access PrEP commodities, and training in counseling skills to enhance service delivery. While training is essential, the high proportion of pharmacists citing the need for logistical support and collaboration with nurses or counselors shows that education alone is insufficient. “Logistical support” may include digital record systems, task-sharing, supply chain support, or access to HIV test kits. Future implementation should explore these needs in detail to tailor interventions effectively. Our study also underscores the importance of gender-sensitive stigma reduction and cultural competency training, especially since women pharmacists were less likely to express interest in providing services to GBMSM. Finally, our spatial analysis reveals the strategic positioning of these pharmacies interested in PrEP delivery in high-risk areas and their potential to serve as critical access points for PrEP delivery to key populations. This study contributes to the literature by demonstrating that many pharmacists in private community pharmacies in Nigeria are willing to provide PrEP services, and their involvement could enhance access for those who need it.

Our findings align with previous studies highlighting pharmacists’ openness to providing PrEP, despite limited prior experience. For example, Broekhuis et al. found that most pharmacists in the United States, although unfamiliar with PrEP protocols, expressed interest in offering the service when adequately supported [27]. Similarly, a study conducted among Canadian pharmacists demonstrated strong readiness to implement PrEP when structured training and collaborative guidelines were available [28]. Regarding how they learnt about PrEP, this study found that professional training (80.9%) was the primary source of PrEP awareness, suggesting a growing integration of HIV prevention education into pharmacy-related professional development. The most commonly reported support need among participants was training on PrEP guidelines (92.1%), confirming earlier findings that identified knowledge gaps in PrEP protocols, medication management, and client counseling [17, 27, 29, 41, 42].

If HIV program planners in Nigeria eventually decide to use private pharmacies as a PrEP delivery platform, it will be essential to carefully consider how pharmacist training will be delivered. We propose two primary approaches. First, a dedicated training course for pharmacy-based PrEP delivery could be developed and integrated into the Continuing Professional Development (CPD) program. Second, PrEP training could be incorporated into the pharmacy school curriculum through clinical pharmacy or pharmacy practice-based courses. An equally important component of this training is the inclusion of gender-sensitive stigma reduction and cultural competency content, particularly given that many individuals in need of PrEP belong to key populations. While overall willingness to serve key populations, such as sex workers and GBMSM, was high, female pharmacists were less likely to offer these services. This gender-based disparity may be influenced by social and cultural norms, stigma, or personal discomfort in addressing the needs of marginalized groups who face systemic barriers to care [43]. Carefully designed training programs are essential to address and reduce these disparities. An important consideration is that the pharmacists who agreed to participate in the survey were already interested in offering PrEP services and had some of the necessary infrastructure in place. The Theory of Planned Behavior (TPB) helps explain their willingness to provide PrEP [33]. Community pharmacists’ positive attitudes toward PrEP delivery suggest that they view it as an effective HIV prevention strategy and recognize its public health value. This shows that they see themselves not only as dispensers of medication but also as accessible healthcare providers. Together with their perceived behavioral control, reflected in their readiness to collaborate with healthcare facilities, their expressed need for training, and having a consultation space, resulted in their willingness to serve those that need PrEP including key populations. This indicates strong potential for successful pharmacy-based PrEP implementation in Nigeria. Evidence from Kenya and the U.S. shows that private pharmacies, when equipped with appropriate tools and referral systems, can safely and effectively deliver PrEP [17, 19, 44, 45]. Overall, these findings show that Nigeria is well-prepared to start testing this new model of delivering PrEP.

Nigeria’s extensive network of retail pharmacies in both urban and peri-urban areas present a significant infrastructural advantage for PrEP delivery and should be leveraged [19, 20]. With the right regulatory support and task-shifting policies, pharmacists could be effectively positioned to address PrEP delivery service gaps, particularly for youths who are often excluded from traditional facility-based care due to stigma or limited operating hours [26, 46–48]. Regulatory reforms that authorize pharmacists to initiate PrEP prescriptions and ensure proper infrastructure—such as private consultation rooms, will be pivotal to this shift [46]. It will also be necessary to formally include pharmacies among the health facilities authorized to offer PrEP so they can receive and dispense donated commodities. Monitoring mechanisms should be established to ensure accountability in the use and distribution of these commodities. Additionally, innovative costing models are essential to ensure the sustainability of such partnerships. For example, in pharmacy-based HIV PrEP delivery, free health commodities, including oral PrEP, post-exposure prophylaxis (PEP), and HIV testing kits, and government-paid HIV clinicians serving as remote oversight could be leveraged, while the cost of pharmacists’ time could be covered through out-of-pocket payments or health insurance.

There are some limitations to this study worth noting. First, the use of purposive sampling may limit the generalizability of the findings. Second, self-reported measures, particularly on sensitive topics related to key populations, may be subject to social desirability bias. Additionally, willingness to provide PrEP does not necessarily translate into actual service delivery unless existing structural and policy barriers are addressed. Fourthly, this study partially relied on publicly available secondary sources for identifying sex work hotspot locations; while useful, these sources may not always be up to date, as official reports often avoid disclosing exact hotspot names for confidentiality reasons. Although the TPB informed the survey design, we did not test the model to examine relationships between its constructs. Future research should assess these relationships and, with larger samples, use multivariable models to better identify factors influencing pharmacists’ willingness to deliver PrEP. Finally, we did not record the number of pharmacists who declined participation, limiting our ability to assess response rates and potential participation bias.

## Conclusion

Community pharmacists in Nigeria demonstrate strong readiness to deliver PrEP and contribute meaningfully to the expansion of HIV prevention services. Their proximity to sex work hotspots positions them as valuable partners in reaching populations in need of PrEP. However, successful implementation will require targeted investments in capacity-building, particularly training that addresses clinical guidelines, cultural competence, and gender-sensitive stigma reduction. Logistical support, including access to government or donor-provided PrEP and HIV RDT kits, and integrating pharmacies into supply chains for PrEP commodities, will also be essential. Future implementation studies are needed to assess the fidelity, feasibility, and real-world outcomes of pharmacy-based PrEP delivery models.

## Data Availability

All the data generated or analyzed during this study are available upon request.
